# A technique for preventing clip snagging during the use of a rotating function endoscopic clip

**DOI:** 10.1055/a-2316-9256

**Published:** 2024-06-12

**Authors:** Koichiro Kawano, Mamoru Takenaka, Reiko Kawano, Tomonori Moriguchi, Koutaro Mine, Katsuhisa Nishi, Masatoshi Kudo

**Affiliations:** 1 Department of Gastroenterology, Hyogo Prefectural Awaji Medical Center, Sumoto, Japan; 2 Department of Gastroenterology and Hepatology, Kindai University Faculty of Medicine, Osakasayama, Japan

The EZ clip series (Olympus, Tokyo, Japan) is widely recognized as a useful endoscopic clip with a rotating function 1 2 3 . This highly effective rotation function allows the clip to be oriented correctly relative to the target ( Fig. 1 ). When employing this function, it is crucial to ensure that the coil sheath does not contact the end of the clip, as frictional resistance may impede proper rotation. Thus, separating the coil sheath from the clip is necessary before initiating rotation. The clip is then rotated to the proper angle and then retracted until the clip stopper contacts the coil sheath to allow for clipping. However, excessive bending of the endoscope during angle adjustment may cause the tip of the clip to collide with the coil sheath, leading to clip snagging ( Fig. 2 ) and, consequently, imprecise clipping ( Fig. 3 ).

**Fig. 1:**
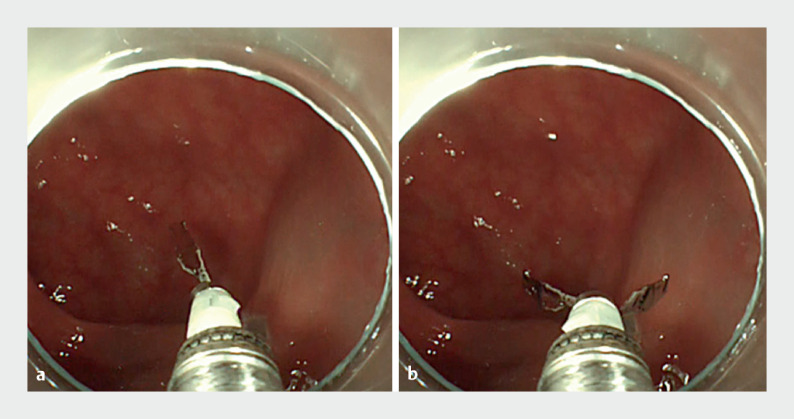
The EZ clip series (Olympus, Tokyo, Japan) is widely recognized as a useful endoscopic clip with a rotating function. This highly effective rotation function allows the clip to be oriented correctly relative to the target.

**Fig. 2:**
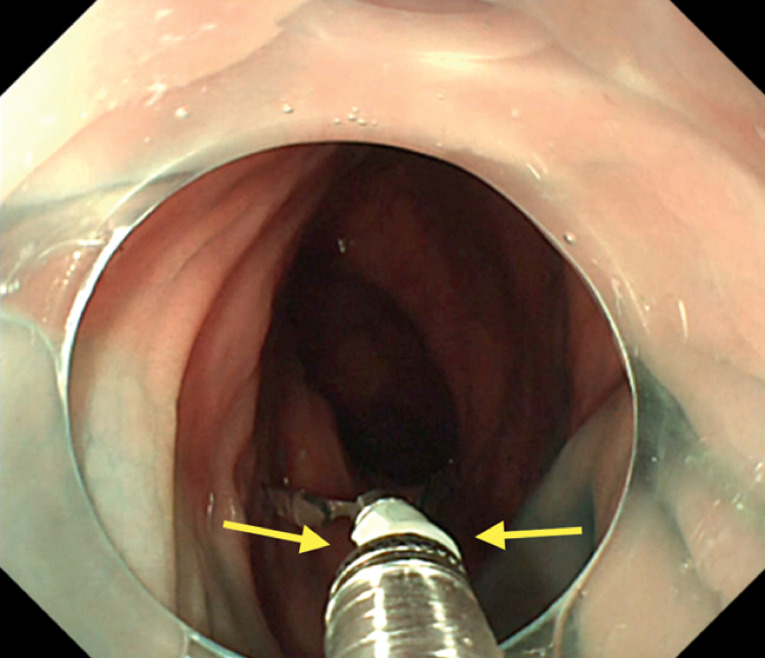
The clip snagging phenomenon. Excessive bending of the endoscope during angle adjustment may cause the tip of the clip to collide with the coil sheath (yellow arrow), leading to clip snagging.

**Fig. 3:**
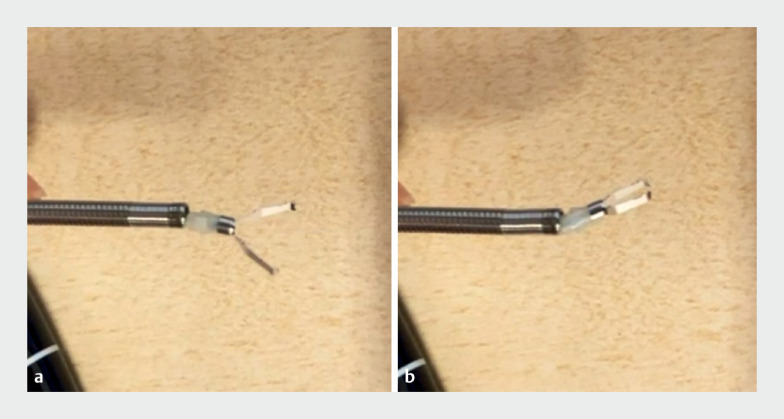
**a** , **b** The clip snagging phenomenon results in imprecise clipping.

Herein, we report a technique for preventing clip snagging during the use of a rotating function endoscopic clip.

The technique was applied to a 67-year-old man undergoing endoscopic submucosal dissection for a 0-IIa+IIc lesion in the sigmoid colon. The EZ clip was employed to suture the wound; however, strong bending of the endoscope tip resulted in clip snagging. To resolve this, the deployed clip and coil sheath were gently retracted into the scope channel while misaligned. The snagging was eliminated after several insertions and withdrawals in the scope channel, as the axis realigned with the coil sheath ( Fig. 4 ). This allowed for clipping without altering the clip’s position or the preset angle ( Fig. 5 , Video 1 ). The patient had a good postoperative course and was discharged on the fourth postoperative day.

**Fig. 4:**
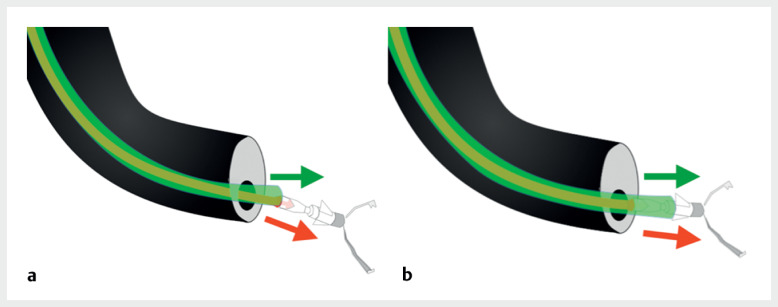
**a** , **b** The deployed clip and coil sheath were gently retracted into the scope channel while misaligned, to resolve the clip snagging phenomenon. The snagging was eliminated after several insertions and withdrawals in the scope channel, as the axis realigned with the coil sheath.

**Fig. 5:**
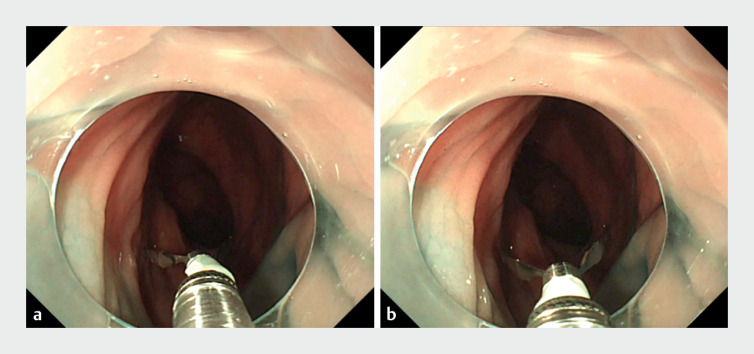
**a** The clip snagging phenomenon. **b** The clip snagging phenomenon was resolved by axis correction.

Technique for preventing clip snagging during the use of a rotating function endoscopic clip for the successful closure of ulcers or effective hemostasis.Video 1

Despite its simplicity, this technique is instrumental in preventing clip snagging with a rotating function endoscopic clip, potentially leading to the successful closure of ulcers and effective hemostasis.

Endoscopy_UCTN_Code_TTT_1AO_2AO

## Notes

Endoscopy E-Videos https://eref.thieme.de/e-videos
*E-Videos* is an open access online section of the journal *Endoscopy* , reporting on interesting cases and new techniques in gastroenterological endoscopy. All papers include a high-quality video and are published with a Creative Commons CC-BY license. Endoscopy E-Videos qualify for HINARI discounts and waivers and eligibility is automatically checked during the submission process. We grant 100% waivers to articles whose corresponding authors are based in Group A countries and 50% waivers to those who are based in Group B countries as classified by Research4Life (see: https://www.research4life.org/access/eligibility/ ). This section has its own submission website at https://mc.manuscriptcentral.com/e-videos .
